# Factors influencing COVID-19 vaccine uptake among vulnerable communities in Texas: Perceptions of 2-1-1 helpline callers^☆^

**DOI:** 10.1016/j.vaccine.2025.127554

**Published:** 2025-08-05

**Authors:** Lara S. Savas, Paula Cuccaro, Kehe Zhang, Rodrigo Hernandez, Meghan E. Haffey, Belinda Reininger, Anais Mendiola, Olivia Dominguez, Maribel Sifuentes, Cici Bauer, Maria E. Fernandez

**Affiliations:** a Department of Health Promotion and Behavioral Sciences, Center for Health Promotion and Prevention Research, The University of Texas Health Science Center at Houston, School of Public Health, Houston, TX, United States; b Department of Biostatistics and Data Science, Center for Spatial-Temporal Modeling for Applications in Population Sciences, The University of Texas Health Science Center at Houston, Houston, TX, United States; c Department of Health Promotion and Behavioral Sciences, Hispanic Health Research Center, The University of Texas Health Science Center at Houston School of Public Health, Brownsville Regional Campus, Brownsville, TX, United States

**Keywords:** COVID-19, SARS-CoV-2, Vaccine, underserved adults, survey, Psychosocial factors

## Abstract

**Background:**

Vaccination effectively and safely prevents serious illness from coronavirus disease 2019 (COVID-19). This study examines the complex factors associated with vaccination uptake among economically disadvantaged adults calling the Texas/United Way 2-1-1 helpline. Results informed the development of strategies to increase COVID-19 vaccine uptake among vulnerable populations disproportionately impacted by COVID-19-related morbidity and mortality.

**Material and methods:**

We conducted a cross-sectional survey among 2-1-1 adult callers (May 2021–September 2022). The survey examined demographic, sociocultural, healthcare access, and psychosocial factors, as well as mistrust in health agencies. We used multivariable logistic regression models to examine factors associated with vaccination uptake, and ordinal regression models to examine factors associated with psychosocial constructs significantly related to vaccination outcomes.

**Results:**

The majority of respondents were from low-income households, had low education attainment, and were minorities. Among the 509 surveys, 67.2 % of participants reported vaccine uptake. Multivariable logistic regression analyses indicated significantly higher odds of vaccination among adults 50–64 years and 65 years and over (OR: 4.5, 95 % CI: 1.7–11.7 and OR: 7.2, 95 % CI: 1.8–28; *P <* 0.01). Respondents with higher scores on perceived susceptibility (OR: 1.4, 95 % CI: 1.0–1.8, *p* = 0.02), perceived safety of the vaccine (OR: 2.9, 95 % CI: 1.9–4.3, *p <* 0.01) and beliefs about vaccine effectiveness against COVID-19 (OR: 1.5, 95 % CI: 1.1–2.0, *p* = 0.02) and serious illness and hospitalization (OR: 1.4, 95 %: 1.0–1.8, p = 0.02) were significantly more likely to have received a vaccination.

**Conclusions:**

Our results indicate that among the sample of economically vulnerable 2-1-1 callers from primarily underserved minority groups, perceived vaccine safety and effectiveness (against COVID-19, serious illness, and hospitalization), and perceived susceptibility to COVID-19 are important factors associated with vaccine uptake. These findings underscore the importance of designing interventions to increase vaccination that address these constructs with powerful messages and strategies.

## 1. Introduction

In the United States (U.S.), over 6.7 million people have experienced a COVID-19-related hospitalization and nearly 1.2 million people have died from COVID-19-related causes as of August 2024 [1,2]. The elderly, individuals with comorbidities, those from low-income communities, and historically underserved minorities have experienced higher COVID-19-related severe disease, hospitalization, and mortality rates [3,4]. COVID-19 vaccines, first released in December 2020, are among the most effective measures to prevent COVID-19 infection [5,6]. Despite growing evidence regarding vaccine effectiveness, safety, and benefits to prevent post-COVID conditions referred to as long COVID [7-9], low vaccination rates persist among uninsured, low-income, and ethnic and racial minority groups (i.e., American Indian or Alaska Native, non-Hispanic Black, and Hispanic), compared with higher income, insured and non-Hispanic White and Asian subgroups [10-13].

Multiple factors contribute to vaccine hesitancy, recently named one of the top ten threats to public health by the World Health Organization [14]. In the U.S., perceptions of racial discrimination, mistrust of governmental public health agencies, and the presence of misinformation or disinformation about the efficacy and safety of the COVID-19 vaccine has contributed to greater vaccine hesitancy in some populations [15-19]. Additionally, political beliefs are associated with COVID-19 vaccination behavior [20,21]. More research is needed to understand the complex psychosocial and sociocultural factors related to COVID-19 vaccination behaviors to inform future strategies aimed at increasing vaccination acceptance, particularly among subgroups with a higher burden of COVID-19-related morbidity and mortality.

The objective of this study was to examine factors associated with COVID-19 vaccination uptake among adults from lower-income and underserved minority groups in Texas. Findings from this study will inform future interventions aimed at increasing COVID-19 vaccine uptake in vulnerable populations.

## 2. Material and methods

### 2.1. Setting and study design

As part of the CDC’s Vaccine Confidence Network, the Center for Health Promotion and Prevention Research (a CDC-funded Prevention Research Center at the UTHealth Houston School of Public Health) collaborated with the United Way 2-1-1 helpline call center to design and implement a survey of 2-1-1 helpline callers to explore their COVID-19 vaccination behaviors and beliefs. The survey was conducted from May 2021 through September 2022. The 2-1-1 helpline is a nationally designated call center that connects low-income and minority callers with needed health and social services [22]. The 2-1-1 Information and Referral specialists (heretofore referred to as 2-1-1 specialists) connect callers to resources in response to expressed needs including assistance with utilities, rent, mortgage, food, transportation, public services, and medical care assistance. The 2-1-1 Texas/United Way helpline is one of the largest in the nation, connecting over 1.25 million Texans to needed services in 2023 [22]. The purpose of this cross-sectional survey was to examine the demographic, economic, sociocultural, psychosocial, health-related, and healthcare utilization-related factors associated with COVID-19 vaccine uptake. The goals were to use this information to inform the development of an intervention to increase COVID-19 vaccine uptake among vulnerable groups residing in the Greater Houston Area and the Lower Rio Grande Valley. This study was reviewed and approved by the UTHealth Houston Committee for the Protection of Human Subjects (IRB # HSC-SPH-21-0633).

### 2.2. Participants

To be eligible for participation in the survey, callers had to be 18 years of age and older, residing in Harris or Cameron counties in Texas, English or Spanish speakers, and agree to participate as indicated by their provided informed consent. Callers in crisis were excluded.

### 2.3. Recruitment and data collection

**Fig. 1.**
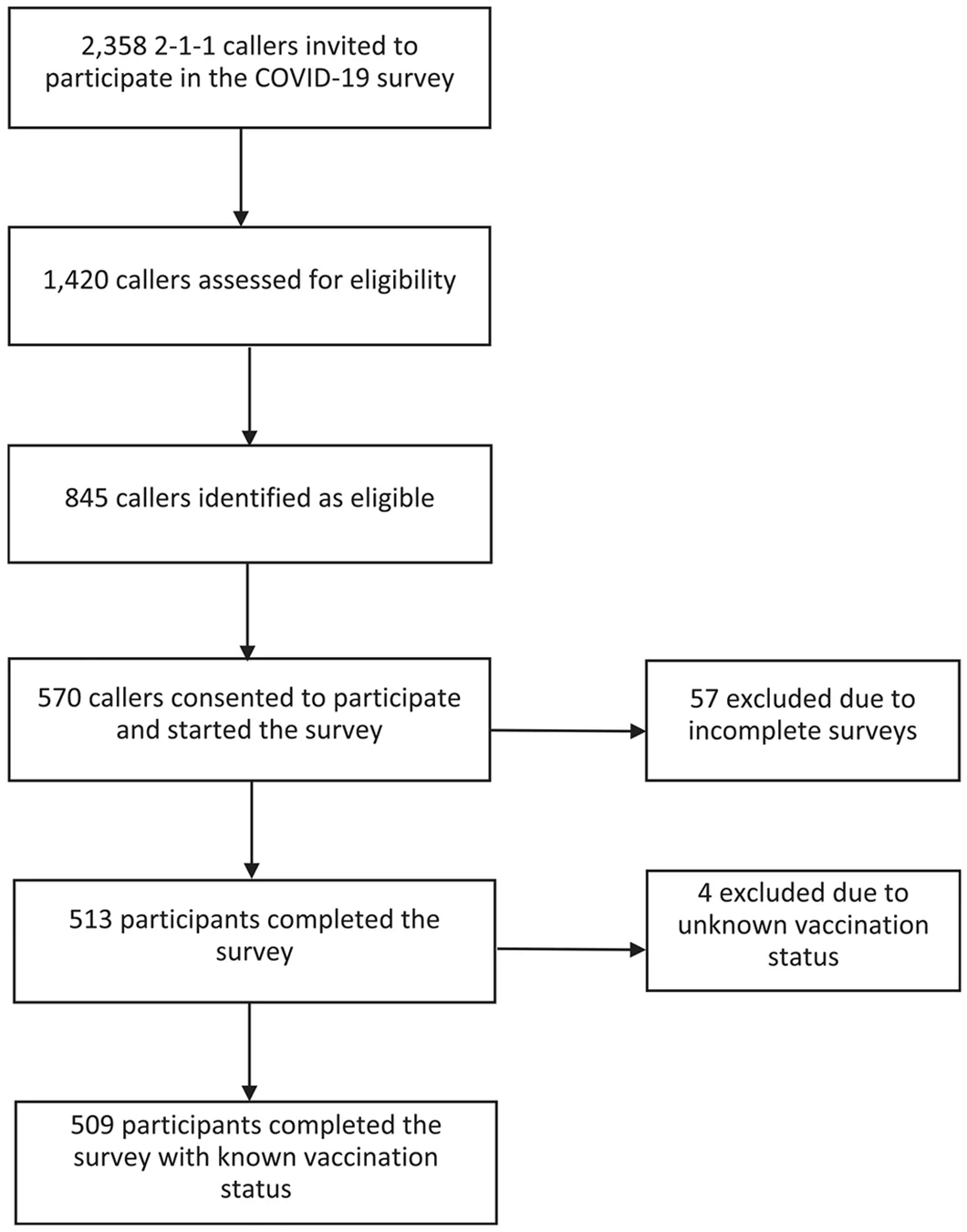
Study Participant Flowchart: Recruitment, Eligibility, and Inclusion of 2-1-1 Callers, April 2022-September 2022.

The UTHealth Houston SPH research team trained three 2-1-1 specialists to conduct the survey in English and one bilingual 2-1-1 specialist to conduct it in English and Spanish. After addressing the callers’ initial reason for calling the 2-1-1 helpline, 2-1-1 specialists invited callers to complete the eligibility questions and administered the survey to eligible callers who provided informed consent. All responses were recorded directly by the specialists in the REDCap-based survey forms, which included scripts and skip patterns to assist in recruitment and verbal survey administration. The survey included 112 items and took approximately 30 min to complete. Participants received $15 gift cards as compensation for their time. Fig. 1 summarizes the recruitment conducted between April 2022 and September 2022. A total of 509 eligible participants completed the survey, including vaccination status, during the time period.

### 2.4. Measures

Measures for psychosocial and individual-level determinants of COVID-19 vaccination behaviors to be assessed were informed by the Health Belief Model and Social Cognitive Theory and a literature review aimed at identifying complex cultural, social, structural, and behavioral factors associated with COVID-19 vaccine uptake [15,23-26]. The CDC COVID-19 Vaccine Confidence Rapid Community Assessment Guide also served as a source for survey measures including items to assess trust in public health agencies, confidence in vaccine effectiveness, perceived vaccine safety, perceived vaccine efficacy, perceived responsibility to be vaccinated, and self-efficacy of getting a COVID-19 vaccine [27].

#### 2.4.1. Primary outcome

COVID-19 vaccination uptake was determined by response to the question *Have you received at least one dose of a COVID-19 vaccine?* Participants answered this question with either “yes” or “no”.

#### 2.4.2. Demographic and healthcare utilization variables

Participant’s demographics (e.g., age, sex, racial/ethnicity, education, income,), sociocultural (e.g., language and political affiliation), health-related characteristics, and health care utilization factors (e.g., health insurance) are described in Table 1. Age was collected as a continuous variable and then categorized into four groups. Race and ethnicity were collected separately and later classified into four groups: non-Hispanic Black or African American, Hispanic or Latino, non-Hispanic White, and Others (including American Indian or Alaska Native, Asian, Multiracial, and Native Hawaiian or Other Pacific Islander). Sex was categorized as male, female and other. We assessed chronic health conditions with one item: *Do you have a chronic health condition*? with “yes” or “no” response options. Perceived access to vaccination services was assessed with the questionnaire item, *How difficult would it be/was it for you to get a COVID-19 vaccine?*

Individual psychosocial and trust-related measures are included in Table 2. Perceived susceptibility assessed participants’ perceived likelihood of developing COVID-19 in the future using a five-item measure that included questions such as *You are at risk for getting COVID-19.* Responses were scored using a 5-point Likert-like scale ranging from 1 (strongly disagree) to 5 (strongly agree). Perceived severity was assessed using a seven-item measure, including four items to assess perceived consequences of COVID-19 infection (e.g., *Getting COVID-19 has severe negative consequences*), scored using a 5-point Likert-like scale ranging from 1 (strongly disagree) to 5 (strongly agree), one item to assess the perceived seriousness of COVID-19 (*How serious do you think COVID-19 is?)* scored using a 5-point scale ranging from 1 (harmless) to 5 (dangerous), and two items to assess the severity of COVID-19 for children and the elderly scored using a 4-point scale ranging from 1 (mild) to 4 (very severe). Items from the perceived susceptibility and severity scales were adapted from measures used in previous research investigating psychosocial determinants of COVID-19 preventive behaviors, COVID-19 vaccine uptake, and HPV vaccine uptake [28-30]. Single items assessing trust in public health agencies, confidence in vaccine effectiveness (i.e., perceived effectiveness against serious illness or hospitalization and perceived vaccine effectiveness against COVID-19), perceived vaccine safety, perceived vaccine efficacy, perceived responsibility, and self-efficacy of getting a COVID-19 vaccine were taken from the CDC COVID-19 Vaccine Confidence Rapid Community Assessment Guide [27].

### 2.5. Statistical analysis

Participants were divided into vaccinated and unvaccinated groups, based on the COVID-19 vaccination primary outcome (yes or no). Descriptive statistics were summarized on the demographic, sociocultural, and health-related characteristics, as well as their healthcare utilization enabling characteristics (e.g., socioeconomic factors), and compared between vaccinated and unvaccinated groups. Additionally, we compared survey responses regarding COVID-19 vaccine attitudes, beliefs, perceptions of COVID-19 susceptibility and severity, and confidence in the COVID-19 vaccine between vaccinated and unvaccinated groups. The multi-item constructs perceived susceptibility and perceived severity were summarized as an average score ranging from 1 (strongly disagree) to 5 (strongly agree). To assess differences between the vaccinated and unvaccinated groups, we tested the association between each construct and vaccination status. We used chi-square or Fisher exact tests for categorical variables and two-sample *t*-tests for continuous variables. All tests were two-sided, with a statistically significant level set at 0.05.

To assess the likelihood of getting vaccinated, we conducted a multivariable logistic regression analysis with vaccination status (yes or no) as the outcome variable. The model included variables found to be significant (*p <* 0.05) in Tables 1 and 2 based on chi-square or two-sample *t*-test. Perceived susceptibility and severity and perceived effectiveness against serious illness or hospitalization were scored from 1 to 5, while trust in public health agencies, perceived safety of the vaccine, and perceived vaccine effectiveness against COVID-19 were scored from 1 to 4. The model was further adjusted for key demographic and socioeconomic variables, including age group, sex, race/ethnicity, household composition, and education level. To account for medical history, we examined chronic health conditions. We excluded variables with missingness greater than 10 % to avoid any bias introduced in the model. We used the variance inflation factor to test for multicollinearity among the explanatory variables included in the multiple logistic regression model. In addition, we examined potential interaction effects between 1). trust in public health agencies and demographic variables and 2). chronic disease conditions and demographic variables based on theoretical relevance and prior literature. Interaction terms were tested and retained in the final model only if they improved model fit as evaluated by likelihood ratio tests.

Further, for each psychosocial construct that was identified as significantly associated with vaccination status in the multivariable logistic regression, we conducted ordinal logistic regression models to assess their relationships with demographic and socioeconomic variables. For each of the multi-item constructs (perceived susceptibility of COVID-19 and severity of COVID-19), we computed the average index score for the items within each construct on a scale from 1 (Strongly Disagree) to 5 (Strongly Agree). We subsequently binned the scores to create an ordinal variable with bin size equal to 1 using the following intervals: Very Low [1,2), Low [2,3), High [3, 4), and Very High [4, 5]. The association between the contributing factors was reported as odds ratio (OR) with 95 % confidence intervals (95 % CI). All data processing and analysis were performed using R (version 4.2.1) in R Studio (version 2023.12.1 + 403) [31].

## 3. Results

Fig. 1 presents the recruitment of 2-1-1 callers. Out of the 2358 invited 2-1-1 callers, 509 participants completed the survey (April 2022–September 2022). The study sample primarily comprised participants who identify as female (80.6 %), identify with a racial or ethnic underserved minority group, reside in low-income households and have limited health care coverage (Table 1). Most participants were 30–49 years old (47 %) and had chronic disease conditions (44.6 %). Among the 509 participants, 98 % completed the interviews in English, and 2 % in Spanish. Using demographic information collected during eligibility screening among consenting eligible callers, we examined age group differences between participants and non-participants. A chi-square test indicated no significant differences in age distribution (*p >* 0.05). However, due to substantial missing data on sex and race/ethnicity among non-participants, we were unable to assess differences on those variables.

Overall, 67.2 % of respondents reported COVID-19 vaccination uptake. Table 1 summarizes and compares the respondents’ demographic, sociocultural, and health-related characteristics, as well as healthcare-enabling characteristics by vaccination status. Unadjusted analysis indicates that older age, living in a household with elderly aged 65 years and older, and lower income, were all associated with having received at least one COVID-19 vaccine. Higher vaccination rates were found among Democrats (79.4 %) compared with Republican and Independents (58.6 % and 54.8 %, respectively; *p <* 0.001). Adults reporting a chronic health condition had a significantly higher vaccination rate than those with no chronic conditions (75.8 % vs. 60.1 %; p *<* 0.001). Among healthcare utilization enabling factors, household income level and perceived access to COVID-19 vaccination services were significantly related to vaccine uptake (Table 1).

Table 2 shows significant differences in COVID-19 vaccine attitudes and beliefs between vaccinated and unvaccinated participants, across all psychosocial constructs, except self-efficacy of getting a COVID-19 vaccine. The variance inflation factor calculated for each variable included was less than 2, indicating little multicollinearity in the regression model.

Table 3 presents the multivariable logistic regression analysis of factors associated with COVID-19 vaccination among 2-1-1 callers. Older age groups had significantly increased odds of vaccination; however, no significant associations were found for sex, race/ethnicity, education, health insurance, COVID-19 infection history, or chronic health conditions. Among psychosocial factors, higher perceived susceptibility (OR: 1.4, 95 % CI: 1.0–1.8, *p* = 0.02), greater confidence in vaccine effectiveness against COVID-19 (OR: 1.5, 95 % CI: 1.1–2.1, *p <* 0.01) and serious illness and hospitalization (OR: 1.4, 95 %: 1.0–1.6, p = 0.02), and perceived safety of the vaccine (OR: 2.9, 95 % CI: 1.9–4.3, p < 0.01) were significantly associated with higher odds of being vaccinated. Other factors, such as perceived severity, trust in public health agencies, and self-efficacy to get vaccinated, were not significant predictors. Of note, while significant in bivariate analyses, the social responsibility factor was excluded from the multivariable logistic regression model due to concerns of multicollinearity. Specifically, the variable had high correlations with four other psychosocial constructs in our model (*r >* 0.5). To avoid redundancy and instability in coefficient estimates, we excluded this variable from the final model. To assess whether the associations between key psychosocial factors and vaccination status varied by subgroup, we tested interaction effects between demographic variables (including age, education, income, and health insurance status) and both chronic disease and trust in public health agencies. None of the interaction effects reached statistical significance, and likelihood ratio tests indicated that inclusion of these terms did not improve overall model fit. As a sensitivity analysis, we included political affiliation in the model with “missing” and “prefer not to answer” as a separate category. It was not significantly associated with vaccination status and did not meaningfully change the results for other variables.

Following the multivariable logistic regression, we used ordinal logistic analyses to examine socio-demographic subgroups associated with the attitudinal factors significantly related to vaccination uptake (as identified by the multivariable logistic regression in Table 3). The psychosocial constructs examined in the ordinal analyses included perceived susceptibility to COVID-19 (examined as a categorical variable: Very Low, Low, High, and Very High), perceived vaccine effectiveness against getting COVID-19, perceived safety of the COVID-19 vaccine, and perceived vaccine effectiveness against serious illness and hospitalization. Table 4 provides the ordinal logistic regression results, revealing that older age groups were significantly more likely to perceive the vaccine as both safe and effective. Higher education attainment groups were significantly less likely to perceive the vaccine as safe. Participants with chronic diseases were more likely to have confidence in vaccine effectiveness.

## 4. Discussion

Among the 2-1-1 survey participants, comprising primarily female (80.6 %), under-represented minorities (85.4 %), and people living in a low-income household (77.8 %), overall 66.7 % reported obtaining a COVID-19 vaccine (May 2021–September 2022), with no significant differences by race/ethnicity. In the adjusted analysis, across all characteristics, adults aged 50 years and older were more likely to have been vaccinated. While other studies conducted among U.S. adults have found a significant relationship between medical mistrust and COVID-19 vaccination outcomes [15,17,18], in our adjusted analysis, the relationship between trust in public health agencies that recommend COVID-19 vaccines and COVID-19 vaccine was not significant, likely due to adjustment by other factors in the multivariable analysis, such as demographics, education, confidence in vaccine safety and effectiveness, and perceived susceptibility to COVID-19 infection. Likewise, while political affiliation (Democrat and Independent compared with Republican) has been associated with higher COVID-19 vaccination rates by multiple studies in the U.S. [20,21], the relationship with vaccination outcome was reduced after adjusting for demographic, education, and psychosocial constructs in our study.

In adjusted analysis, having a chronic health condition was not significantly associated with COVID-19 vaccination uptake, despite a higher risk of severe illness. Among participants aged 65 and older, 78 % had a chronic disease (70 % in the 50–64 age group), whereas only 20 % of those aged 18–29 and 33 % of those aged 30–49 reported having a chronic disease. Since variation in vaccination status was largely explained by age in the model, the effect of chronic disease on vaccination status was diminished in our adjusted model. Additionally, those who reported having a previous COVID-19 infection did not have a higher odds of vaccination. This could be due to people with a history of COVID-19 believing they had a natural immunity, thus opting not to get vaccinated afterward, as reported previously [32], despite evidence that indicates getting the vaccine offers added protection against COVID-19 and long COVID for those who have had a previous COVID-19 infection [33-35]. This finding, coupled with emerging evidence regarding immunity developed through a combination of COVID-19 infection and vaccination, suggests a need to incorporate messages about benefits of COVID-19 vaccination in the context of COVID-19 infection history.

Ordinal logistic regression analysis results, which examined the relationship between demographic and socioeconomic characteristics and attitudes significantly related to vaccine outcomes (i.e., perceived susceptibility of COVID-19, perceived vaccine safety, perceived vaccine effectiveness against COVID-19, and perceived vaccine effectiveness against serious illness or hospitalization), indicated that no socioeconomic or demographic factors were associated with perceived susceptibility to COVID-19. Older age was the only characteristic associated with increased odds of perceiving the COVID-19 vaccine is safe. While older age and having a chronic disease were associated with increased perceived vaccine effectiveness against COVID-19, older age and being male were associated with perceived vaccine effectiveness against severe illness and hospitalization. These findings indicate increased focus on messaging vaccine safety and effectiveness is needed, particularly in younger adults [16,28,36]. It is noteworthy that chronic disease status did not increase vulnerable adults’ perceived susceptibility to COVID-19, despite evidence that having a chronic disease predisposes a person to more severe COVID-19 outcomes [37-39]. The relationship, however, may have been reduced by adjustment for age. The findings indicate a need for public health interventions to develop effective messages regarding COVID-19 susceptibility to promote vaccine uptake, particularly among vulnerable groups in the younger age groups, and those with chronic disease conditions.

This study has some potential limitations. Study findings are generalizable to vulnerable populations, particularly women, and those seeking support to meet their basic needs, such as social and health services (e.g., people calling 2-1-1 helplines), and may not be representative of a wider population of persons in vulnerable communities. Another limitation is the reliance on self-reported data, which introduces the potential threat of memory recall and social desirability bias; however, we may have minimized the social desirability bias threat by engaging expert interviewers (as discussed above). Volunteer bias (self-selection) is another potential limitation. While we found no self-selection bias differences by age distribution (*p >* 0.05), due to substantial missing data on sex and race/ethnicity among non-participants, we were unable to assess potential differences between participants and non-participants by these characteristics, or other unmeasured factors. Callers who agreed to participate may have had different characteristics compared to those who declined (e.g., those who declined the survey may have felt more urgency to resolve the issue they initially called 2-1-1 about and declined to complete the survey to focus on their reason for calling), which could affect the generalizability of the findings. Additionally, only one 2-1-1 specialist was bilingual, which limited inclusion of Spanish speakers. However, because the 2-1-1 callers are mostly low-income and all called 2-1-1 for help meeting basic needs, the participants remain representative of groups experiencing economic vulnerability in the study region.

A major study strength was the 2-1-1 helpline collaboration in conducting the survey, which facilitated surveying traditionally hard-to-reach populations disproportionately affected by COVID-19. The 2-1-1 specialists were also able to seamlessly serve callers and address their reason for calling 2-1-1 before inviting them to the study and completing the survey, thus establishing trust with the caller before conducting the survey. The 2-1-1 helpline setting and ability to leverage the 2-1-1 specialists’ expertise in cultural competency and understanding of the population they serve also helped to ensure the 2-1-1 callers were engaged with an ethical and culturally sensitivity approach. The 2-1-1 specialists’ communication expertise and understanding of the needs of the population they serve was a strength that potentially increased participation, helping enhance external validity, as well as potentially minimizing social desirability bias, helping to enhance internal validity.

In conclusion, this study provides important insight into the complex factors influencing vaccination uptake among vulnerable and hard-to-reach groups. In May 2024, an estimated 22.5 % of adults in the U.S., and 15.6 % of adults living below the poverty level reported receiving the updated COVID-19 vaccine after its introduction on September 14, 2023 [40]. The decline of COVID-19 vaccine uptake underscores the need for research to inform public health campaign messaging and strategies to prioritize the most under-vaccinated and vulnerable groups, as represented in this study. Our study results suggest vaccination campaigns should incorporate strong messages that address vaccine safety and effectiveness (particularly among younger adults), and perceived susceptibility to COVID-19 across all vulnerable adult demographic subgroups. Findings also indicate a need for future studies to understand factors that influence perceived susceptibility to COVID-19 infection, an attitude found significantly related to vaccination uptake in vulnerable adults. These findings can help guide future programs aimed at promoting vaccination, a need evidenced by the persistence of low vaccination rates, particularly among vulnerable populations.

## Figures and Tables

**Table 1:** Characteristics of 2-1-1 callers: A comparison by vaccination status, May 2021 to September 2022 (*N* = 509).

Variables	All Participants N = 509	Vaccinated *N* = 342	Unvaccinated *N* = 167	P-value*
	(Column %)	(Row %)		
**Demographic, Sociocultural, and Health-Related Characteristics**				
**Age Group**				**< 0.001**
*What is your current age?*				
18–29	87 (17.1 %)	37 (42.5 %)	50 (57.5 %)	
30–49	241 (47.3 %)	151 (62.7 %)	90 (37.3 %)	
50–64	112 (22.0 %)	91 (81.2 %)	21 (18.8 %)	
65 and greater	69 (13.6 %)	63 (91.3 %)	6 (8.7 %)	
**Sex identity**				0.098
Female	410 (80.6 %)	272 (66.3 %)	138 (33.7 %)	
Male	95 (18.7 %)	69 (72.6 %)	26 (27.4 %)	
Others	4 (0.8 %)	1 (25 %)	3 (75 %)	
**Race/Ethnicity** **				0.386
Non-Hispanic Black or African American	329 (64.6 %)	221 (67.2 %)	108 (32.8 %)	
Hispanic or Latino	106 (20.8 %)	75 (70.8 %)	31 (29.2 %)	
Non-Hispanic White	48 (9.4 %)	31 (64.6 %)	17 (35.4 %)	
Others	20 (3.9 %)	10 (50 %)	10 (50 %)	
Prefer not to answer/Missing	6 (1.2 %)	5 (83.3 %)	1 (16.7 %)	
**Household composition**				**< 0.001**
*Do you have children in your home under 18 years old?*				
Yes	250 (49.1 %)	146 (58.4 %)	104 (41.6 %)	
No	259 (50.9 %)	196 (75.7 %)	63 (24.3 %)	
*Do you have someone in your household who is 65 years or older?*				**< 0.001**
Yes	95 (18.7 %)	79 (83.2 %)	16 (16.8 %)	
No	412 (80.9 %)	262 (63.6 %)	150 (36.4 %)	
Missing	2 (0.4 %)	1 (50 %)	1 (50 %)	
**Language spoken at home other than English**				0.178
*Do you speak a language other than English at home?*				
Yes	100 (19.6 %)	75 (75 %)	25 (25 %)	
No	397 (78.0 %)	259 (65.2 %)	138 (34.8 %)	
Prefer not to answer/Missing	12 (2.4 %)	8 (66.7 %)	4 (33.3 %)	
**Education Attainment**				0.725
*What is the highest level of education you have achieved outside or in the United States?*				
No high school diploma	101 (19.8 %)	70 (69.3 %)	31 (30.7 %)	
High school graduate or GED completed	162 (31.8 %)	102 (63 %)	60 (37 %)	
Some college level/Technical/ Vocational degree	191 (37.5 %)	131 (68.6 %)	60 (31.4 %)	
Bachelor’s degree and above	52 (10.2 %)	37 (71.2 %)	15 (28.8 %)	
**Political Affiliation**				**< 0.001**
*Generally speaking, do you usually think of yourself as a Republican, Democrat, Independent, or something else?*				
Democrat	199 (39.1 %)	158 (79.4 %)	41 (20.6 %)	
Republican	29 (5.7 %)	17 (58.6 %)	12 (41.4 %)	
Independent	146 (28.7 %)	80 (54.8 %)	66 (45.2 %)	
Something else	67 (13.2 %)	45 (67.2 %)	22 (32.8 %)	
Prefer not to answer/Missing	68 (13.4 %)	42 (61.8 %)	26 (38.2 %)	
**COVID-19 History**				0.268
*Have you ever tested positive for COVID-19?*				
Yes	181 (35.6 %)	116 (64.1 %)	65 (35.9 %)	
No	326 (64.0 %)	226 (69.3 %)	100 (30.7 %)	
Missing	2 (0.4 %)	0 (0 %)	2 (100 %)	
**Chronic Health Conditions**				**< 0.001**
*Do you have a chronic health condition?*				
Yes	227 (44.6 %)	172 (75.8 %)	55 (24.2 %)	
No	278 (54.6 %)	167 (60.1 %)	111 (39.9 %)	
Not sure	4 (0.8 %)	3 (75 %)	1 (25 %)	
**Health Care Utilization Enabling Characteristics**				
**Total Household Income**				**0.004**
*In 2021, what was your total household income before taxes?*				
Less than $25,000	278 (54.6 %)	203 (73 %)	75 (27 %)	
$25,000 - $49,999	118 (23.2 %)	76 (64.4 %)	42 (35.6 %)	
$50,000 and above	27 (5.3 %)	18 (66.7 %)	9 (33.3 %)	
Prefer not to answer/Missing	86 (16.9 %)	45 (52.3 %)	41 (47.7 %)	
**Health Insurance Status**				0.811
*Do you currently have health insurance?*				
Yes	354 (69.5 %)	241 (68.1 %)	113 (31.9 %)	
No	152 (29.9 %)	99 (65.1 %)	53 (34.9 %)	
Not sure/Missing	3 (0.6 %)	2 (66.7 %)	1 (33.3 %)	
**Provider Recommendation**				0.143
*Has a doctor, nurse or other healthcare provider ever recommended that you get the COVID-19 vaccine?*				
Yes	293 (57.6 %)	207 (70.6 %)	86 (29.4 %)	
No	214 (42.0 %)	134 (62.6 %)	80 (37.4 %)	
Don’t Know	2 (0.4 %)	1 (0.3 %)	1 (0.6 %)	
**Perceived Access to Vaccination Service**				**0.039**
*How difficult would it be/was it for you to get a COVID-19 vaccine?*				
Not at all difficult	363 (71.3 %)	254 (70 %)	109 (30 %)	
A little difficult	31 (6.1 %)	21 (67.7 %)	10 (32.3 %)	
Somewhat	58 (11.4 %)	39 (67.2 %)	19 (32.8 %)	
Very difficult	54 (10.6 %)	27 (50 %)	27 (50 %)	
Prefer not to answer/Missing	3 (0.6 %)	1 (33.3 %)	2 (66.7 %)	

Note: Column percentages were reported for all participants, while row percentages were provided for groups stratified by vaccination status.

* P-values were derived from Chi-square/Fisher exact tests or two-sample t-tests. Bolded p-values indicate a statistically significant difference in the distribution of the variable between vaccinated and unvaccinated groups.

** Race/Ethnicity was derived from two questions: 1) Are you of Hispanic, Latino/a, or Spanish origin? 2) What is your race?

**Table 2:** COVID-19 vaccine uptake by vaccine-related attitudes, beliefs, and perceptions of COVID-19 susceptibility and severity and confidence in the vaccine, May 2021 to September 2022 (N = 509).

	All participants (N = 509)	Vaccinated (N = 342)	Unvaccinated (N = 167)	P-value*
**Perceived susceptibility to COVID-19** **				**0.001**
Mean (SD)	2.82 (1.13)	2.93 (1.15)	2.59 (1.04)	
Median [Min, Max]	2.80 [1.00, 5.00]	2.80 [1.00, 5.00]	2.40 [1.00, 5.00]	
Missing	8 (1.6 %)	6 (1.8 %)	2 (1.2 %)	
**Perceived severity of COVID-19** **				**0.008**
Mean (SD)	3.91 (0.663)	3.97 (0.627)	3.80 (0.720)	
Median [Min, Max]	4.14 [1.43, 4.71]	4.14 [1.71, 4.71]	4.00 [1.43, 4.71]	
Missing	21 (4.1 %)	15 (4.4 %)	6 (3.6 %)	
**Trust in public health agencies**				**< 0.001**
*How much do you trust the public health agencies that recommend COVID-19 vaccines?*				
Do not trust	110 (21.6 %)	34 (30.9 %)	76 (69.1 %)	
Somewhat trust	197 (38.7 %)	126 (64 %)	71 (36 %)	
Fully trust/Mostly trust	199 (39.1 %)	181 (91 %)	18 (9 %)	
Missing	3 (0.6 %)	1 (33.3 %)	2 (66.7 %)	
**Perceived vaccine effectiveness against getting COVID-19**				
*How important do you think getting a COVID-19 vaccine is to protect yourself against COVID-19?*				**< 0.001**
Not at all important	70 (13.8 %)	12 (17.1 %)	58 (82.9 %)	
A little important/Somewhat important	155 (30.5 %)	79 (51 %)	76 (49 %)	
Very important	279 (54.8 %)	248 (88.9 %)	31 (11.1 %)	
Missing	5 (1.0 %)	3 (60 %)	2 (40 %)	
**Perceived vaccine safety (risk)**				
*How safe do you think a COVID-19 vaccine is for you?*				**< 0.001**
Not at all safe	108 (21.2 %)	18 (16.7 %)	90 (83.3 %)	
Somewhat safe	179 (35.2 %)	117 (65.4 %)	62 (34.6 %)	
Very safe/Completely safe	213 (41.8 %)	202 (94.8 %)	11 (5.2 %)	
Missing	9 (1.8 %)	5 (55.6 %)	4 (44.4 %)	
**Perceived vaccine effectiveness against serious illness or hospitalization**				
*COVID-19 vaccines protect people from getting seriously ill and needing hospitalization.*				**< 0.001**
Disagree/Strongly Disagree	352 (69.2 %)	57 (41.3 %)	81 (58.7 %)	
Neither agree nor disagree	14 (2.8 %)	8 (57.1 %)	6 (42.9 %)	
Agree/Strongly Agree	138 (27.1 %)	273 (77.6 %)	79 (22.4 %)	
Missing	5 (1.0 %)	4 (80 %)	1 (20 %)	
**Social Responsibility**				
*Do you feel you have a responsibility to get vaccinated for COVID-19 to protect others?*				**< 0.001**
Strongly Disagree	62 (12.2 %)	12 (19.4 %)	50 (80.6 %)	
Disagree	95 (18.7 %)	32 (33.7 %)	63 (66.3 %)	
Neither agree nor disagree	6 (1.2 %)	1 (16.7 %)	5 (83.3 %)	
Agree	140 (27.5 %)	107 (76.4 %)	33 (23.6 %)	
Strongly Agree	203 (39.9 %)	187 (92.1 %)	16 (7.9 %)	
Missing	3 (0.6 %)	3 (100 %)	0 (0 %)	
**Self-efficacy of getting COVID-19 vaccine**				0.075
*You can get a COVID-19 vaccine if you want to.*				
Strongly Disagree	17 (3.3 %)	8 (47.1 %)	9 (52.9 %)	
Disagree	13 (2.6 %)	9 (69.2 %)	4 (30.8 %)	
Neither agree nor disagree	0 (0 %)	0 (0 %)	0 (0 %)	
Agree	207 (40.7 %)	130 (62.8 %)	77 (37.2 %)	
Strongly Agree	267 (52.5 %)	190 (71.2 %)	77 (28.8 %)	
Missing	5 (1.0 %)	5 (100 %)	0 (0 %)	

Column percentages were reported for all participants, while row percentages were provided for groups stratified by vaccination status.

* *P*-values are derived from Chi-square/Fisher exact tests or two-sample *t*-tests. Bolded *p*-values indicate a statistically significant difference in the distribution of the variable between vaccinated and unvaccinated groups.

** The multi-item constructs perceived susceptibility and perceived severity were summarized as an average score ranging from 1 (Strongly Disagree) to 5 (Strongly Agree).

**Table 3:** Multivariable logistic regression analysis assessing factors associated with COVID-19 vaccination status among 2-1-1 callers (*N* =463), May 2021–September 2022.

	Odds Ratio (95 % CI)	*P* value
**Age Category**		
18–29	Reference	
30–49	1.7 (0.9, 3.4)	0.12
50–64	**4.5 (1.7, 11.7)**	**<0.01**
65 and greater	**7.2 (1.8, 28)**	**<0.01**
**Sex**		
Female	Reference	
Male	0.9 (0.4, 1.8)	0.68
**Race/Ethnicity**		
Non-Hispanic White	Reference	
Non-Hispanic Black/African American	1.0 (0.4, 2.5)	0.99
Hispanic or Latino	1.7 (0.6, 4.8)	0.34
Others	0.8 (0.2, 3.9)	0.77
**Households with children under 18**		
No	Reference	
Yes	0.9 (0.5, 1.7)	0.73
**Education**		
No high school diploma	Reference	
High school graduate/GED	1.1 (0.5, 2.5)	0.85
Some college level/technical/vocational degree	2.3 (1.0, 5.4)	0.06
Bachelor’s degree	2.6 (0.8, 8.1)	0.10
**Chronic health condition**		
No	Reference	
Yes	1.1 (0.6, 2.1)	0.74
**Attitudes about COVID-19, the vaccine, and trust in public health agencies** *		
Perceived susceptibility	**1.4 (1.0, 1.8)**	**0.02**
Perceived severity	0.7 (0.4, 1.1)	0.09
Trust in public health agencies	1.4 (0.9, 2.0)	0.10
Perceived vaccine effectiveness against getting COVID-19	**1.5 (1.1, 2.0)**	**0.02**
Perceived safety of vaccine	**2.9 (1.9, 4.3)**	**<0.01**
Perceived access (difficulty) to vaccination service	0.9 (0.7, 1.2)	0.47
Perceived vaccine effectiveness against serious illness or hospitalization	**1.4 (1.0, 1.8)**	**0.02**

**Bold:** *p* <0.05.

**Note:** *Records with more* than 10 % missing data were excluded, including political affiliation and household income, *resulting in a final sample size of 463.*

Household with elderly (65+ years) was excluded due to the high correlation with age groups.

* The psychosocial constructs were included in the model as an average score on a continuous scale from 1 to 5.

**Table 4:** Factors associated with perceived susceptibility of COVID-19, safety of COVID-19 vaccine, and confidence in COVID-19 vaccine effectiveness: Results from ordinal logistic regression analysis.

	Perceived Susceptibility of COVID-19 (N* = 490)	Perceived Safety of COVID-19 vaccine (N = 490)	Perceived vaccine effectiveness against COVID-19 (*N =* 490)	Perceived vaccine effectiveness against serious illness or hospitalization (*N* = 487)
**Age Group**				
18–29	Reference	Reference	Reference	Reference
30–49	0.89 (0.56, 1.42)	**1.59 (1.01, 2.52)**	**2.3 (1.44, 3.68)**	**2.1 (1.33, 3.32)**
50–64	1.2 (0.68, 2.09)	**3.77 (2.13, 6.67)**	**4.6 (2.5, 8.46)**	**2.21 (1.26, 3.88)**
65 and older	0.9 (0.48, 1.71)	**5.8 (3.04, 11.05)**	**13.56 (5.66, 32.54)**	**4.77 (2.5, 9.08)**
**Sex**				
Female	Reference	Reference	Reference	Reference
Male	0.72 (0.47, 1.1)	1.21 (0.79, 1.85)	1.08 (0.67, 1.73)	**1.59 (1.03, 2.45)**
**Race/Ethnicity**				
Non-Hispanic White	Reference	Reference	Reference	Reference
Non-Hispanic Black or African American	1.47 (0.84, 2.6)	0.91 (0.51, 1.63)	1.24 (0.67, 2.3)	0.92 (0.52, 1.63)
Hispanic or Latino	1.57 (0.83, 2.97)	0.96 (0.5, 1.85)	1.39 (0.69, 2.8)	1.17 (0.61, 2.23)
Others	1.36 (0.48, 3.88)	0.45 (0.17, 1.17)	0.81 (0.31, 2.12)	1.65 (0.63, 4.33)
**Chronic Disease**				
No	Reference	Reference	Reference	Reference
Yes	1.15 (0.8, 1.65)	0.86 (0.6, 1.25)	**1.53 (1.03, 2.28)**	1.22 (0.84, 1.75)
**Education**				
No high school diploma	Reference	Reference	Reference	Reference
High school graduate	0.69 (0.43, 1.11)	0.72 (0.44, 1.18)	0.93 (0.53, 1.61)	0.94 (0.57, 1.54)
Some college level	0.7 (0.45, 1.11)	**0.57 (0.35, 0.92)**	0.68 (0.4, 1.16)	0.67 (0.41, 1.07)
Bachelor’s degree	0.75 (0.41, 1.38)	0.61 (0.32, 1.17)	0.6 (0.3, 1.2)	**0.48 (0.25, 0.93)**

**Note**: N is the sample size for each ordinal regression model.

## Data Availability

Data will be made available on request.
